# SARS-CoV-2 infection and acute ischemic stroke in Lombardy, Italy

**DOI:** 10.1007/s00415-021-10620-8

**Published:** 2021-05-24

**Authors:** Alessandro Pezzini, Mario Grassi, Giorgio Silvestrelli, Martina Locatelli, Nicola Rifino, Simone Beretta, Massimo Gamba, Elisa Raimondi, Giuditta Giussani, Federico Carimati, Davide Sangalli, Manuel Corato, Simonetta Gerevini, Stefano Masciocchi, Matteo Cortinovis, Sara La Gioia, Francesca Barbieri, Valentina Mazzoleni, Debora Pezzini, Sonia Bonacina, Andrea Pilotto, Alberto Benussi, Mauro Magoni, Enrico Premi, Alessandro Cesare Prelle, Elio Clemente Agostoni, Fernando Palluzzi, Valeria De Giuli, Anna Magherini, Daria Valeria Roccatagliata, Luisa Vinciguerra, Valentina Puglisi, Laura Fusi, Susanna Diamanti, Francesco Santangelo, Rubjona Xhani, Federico Pozzi, Giampiero Grampa, Maurizio Versino, Andrea Salmaggi, Simona Marcheselli, Anna Cavallini, Alessia Giossi, Bruno Censori, Carlo Ferrarese, Alfonso Ciccone, Maria Sessa, Alessandro Padovani, the STROKOVID group

**Affiliations:** 1 Department of Clinical and Experimental Sciences, University of Brescia, P.le Spedali Civili, 1, 25123 Brescia, Italy; 2 Department of Brain and Behavioural Sciences, Statistics and Genomic Unit, University of Pavia, Pavia, Italy; 3 Department of Neurology and Stroke Unit, Carlo Poma Hospital, ASST Mantova, Mantova, Italy; 4 Neurology Unit, Istituti Ospitalieri, ASST Cremona, Cremona, Italy; 5 Department of Neurology, Ospedale San Gerardo, ASST Monza, Monza, Italy; 6 Department of Medicine and Surgery and Milan Center for Neuroscience, University of Milano-Bicocca, Milan, Italy; 7 Vascular Neurology-Stroke Unit, Spedali Civili Hospital, ASST Spedali Civili, Brescia, Italy; 8 Neurology Unit, Ospedale Nuovo, ASST Ovest Milanese, Legnano, Italy; 9 Neurology Unit and Stroke Unit, Department of Neurosciences and Niguarda Neuro Center, ASST Grande Ospedale Metropolitano Niguarda, Milan, Italy; 10 Neurology Unit, Ospedale di Circolo e Fondazione Macchi, ASST Sette Laghi, Varese, Italy; 11 Neurology Unit, Ospedale “A. Manzoni”, ASST Lecco, Lecco, Italy; 12 Emergency Neurology and Stroke Unit, IRCCS Humanitas Clinical and Research Center, Rozzano, Milan, Italy; 13 Department of Neuroradiology, Papa Giovanni XXIII Hospital, ASST Papa Giovanni XXIII, Bergamo, Italy; 14 Department of Neurology, Papa Giovanni XXIII Hospital, ASST Papa Giovanni XXIII, Bergamo, Italy; 15 Neurology Unit, Ospedale “Sant’Anna”, ASST Lariana, Como, Italy; 16 Stroke Unit, IRCCS Fondazione “C. Mondino”, Pavia, Italy

**Keywords:** Stroke, Risk factors, COVID-19, Viral infection

## Abstract

**Objective:**

To characterize patients with acute ischemic stroke related to SARS-CoV-2 infection and assess the classification performance of clinical and laboratory parameters in predicting in-hospital outcome of these patients.

**Methods:**

In the setting of the STROKOVID study including patients with acute ischemic stroke consecutively admitted to the ten hub hospitals in Lombardy, Italy, between March 8 and April 30, 2020, we compared clinical features of patients with confirmed infection and non-infected patients by logistic regression models and survival analysis. Then, we trained and tested a random forest (RF) binary classifier for the prediction of in-hospital death among patients with COVID-19.

**Results:**

Among 1013 patients, 160 (15.8%) had SARS-CoV-2 infection. Male sex (OR 1.53; 95% CI 1.06–2.27) and atrial fibrillation (OR 1.60; 95% CI 1.05–2.43) were independently associated with COVID-19 status. Patients with COVID-19 had increased stroke severity at admission [median NIHSS score, 9 (25th to75th percentile, 13) vs 6 (25th to75th percentile, 9)] and increased risk of in-hospital death (38.1% deaths vs 7.2%; HR 3.30; 95% CI 2.17–5.02). The RF model based on six clinical and laboratory parameters exhibited high cross-validated classification accuracy (0.86) and precision (0.87), good recall (0.72) and F1-score (0.79) in predicting in-hospital death.

**Conclusions:**

Ischemic strokes in COVID-19 patients have distinctive risk factor profile and etiology, increased clinical severity and higher in-hospital mortality rate compared to non-COVID-19 patients. A simple model based on clinical and routine laboratory parameters may be useful in identifying ischemic stroke patients with SARS-CoV-2 infection who are unlikely to survive the acute phase.

**Supplementary Information:**

The online version contains supplementary material available at 10.1007/s00415-021-10620-8.

## Introduction

With the increasing number of confirmed cases of coronavirus disease 2019 (COVID-19), caused by the severe acute respiratory syndrome coronavirus 2 (SARS-CoV-2), and the accumulating clinical data, it is now well established that, in addition to the predominant respiratory symptoms, a significant proportion of COVID-19 patients has extrapulmonary manifestations, including thrombotic complications, myocardial dysfunction and arrhythmia, acute coronary syndromes, acute kidney injury, gastrointestinal symptoms, hepatocellular injury, hyperglycemia and ketosis, neurologic illnesses, such as acute stroke, ocular symptoms, and dermatologic complications [1, 2]. SARS-CoV-2 interacts with cardiovascular system on multiple levels, increasing morbidity in patients with underlying cardiovascular conditions, provoking myocardial injury and dysfunction, and acute cerebrovascular disease, as well [1, 3]. The proposed mechanisms for cerebrovascular events include a hypercoagulable state from systemic inflammation and cytokine storm, postinfectious immune-mediated responses and direct viral-induced endotheliitis or endotheliopathy, potentially leading to angiopathic thrombosis, with viral particles having been isolated from various tissue, including brain tissue [3]. Acute stroke has been reported among the most frequent neurological features of coronavirus viremia, complicating 1.0% to 3.7% of COVID-19 patients, and up to 76.8% of cases with confirmed infection and neurological manifestations, according to the few series reported so far [3]. However, although we now know much more about the biological processes that link SARS-CoV-2 infection to stroke, the characteristics of patients with acute ischemic stroke related to infection, the mechanisms underlying brain ischemia, and the predictors of in-hospital outcome of these patients are not yet fully identified. Furthermore, many currently available data are derived from studies comparing patients with stroke and historical controls [4–6]. Such an approach does not allow to exclude a potential bias since historical controls tend to have less severe strokes than those observed among contemporaneous controls [7–10]. In the present study, we aimed to fill the gap by investigating these issues in the setting of the STROKOVID project, a multicentre study conducted in Lombardy, Northern Italy, one of the largest registries including patients with COVID-19 and acute ischemic stroke that is currently available.

## Methods

### Study group

Data were collected in the setting of a prospective, hospital-based, multicentre study conducted in Lombardy, Northern Italy. Because of the spread of the epidemic, on March 8, 2020, the Lombardy regional government passed a deliberation to reduce to ten the hospitals with catheterization facilities for the treatment of acute ischemic stroke acting as hubs, with the remaining hospitals acting as spokes, on the basis of geographic proximity. Since these ten centers were designated hospitals for transfer of patients from the contiguous catchment area at the early stage of the outbreak, they included all patients who were hospitalized in Lombardy for acute ischemic stroke during the epidemic. The STROKOVID network is a joint initiative of these ten hub centers, which is expected to provide comprehensive information on patients hospitalized for acute ischemic stroke in Lombardy during SARS-CoV-2 outbreak and to address clinical research questions (Supplementary Fig. 1). The present study is a retrospective analysis of data from patients consecutively admitted to the participating hospitals between March 8 and April 30, 2020. All patients with symptoms suggestive of acute stroke were screened for inclusion. Patients were enrolled in the STROKOVID registry if they fulfilled the following criteria: (1) a clinical diagnosis of acute stroke; (2) CT/MRI scan results with no evidence of other causes that might explain the neurological deficits. A case–control study was designed and reported according to STROBE guidelines [11].

### Risk factors

The following conditions were retained as risk factors for acute ischemic stroke: hypertension, diabetes, smoking habit, hypercholesterolemia, body mass index (BMI), atrial fibrillation (AF), history of coronary ischemic heart disease, history of ischemic stroke, chronic obstructive pulmonary disease (COPD), and chronic kidney disease (CKD). We also collected information on use of pre-stroke medications and functional status before stroke occurrence (Supplementary Materials and Methods).

### Clinical assessment

Patients received an initial diagnostic evaluation and treatment based on established guidelines [12]. In particular, those who were deemed eligible, received intravenous thrombolysis or endovascular mechanical thrombectomy or a combined treatment with full-dose intravenous rtPA and “contemporary/as soon as possible” endovascular mechanical thrombectomy. All patients were classified into etiologic subgroups according to the Trial of ORG 10,172 in Acute Stroke Treatment criteria [13] by local investigators at each study center. Stroke severity was scored by the National Institutes of Health (NIH) Stroke Scale [14]. Based on the severity of respiratory impairment, patients with SARS-CoV-2 infection were divided into four groups according to whether there were clinical respiratory symptoms, severity of pneumonia, respiratory failure, shock, or other organ failure [15] (Supplementary Materials and Methods).

### Medical complications

The following conditions were retained as in-hospital medical complications of acute stroke: sepsis and septic shock, secondary infection, acute respiratory distress syndrome (ARDS), acute kidney injury, acute cardiac injury, coagulopathy, hypoproteinaemia, recurrent ischemic stroke, and symptomatic intracranial hemorrhage (Supplementary Materials and Methods).

### Laboratory procedures

Laboratory confirmation of SARS-CoV-2 infection was made by RT PCR procedure on throat-swab and nasopharyngeal specimens [16] in all patients admitted to the participating hospitals. In case of high clinical suspicion of SARS-CoV-2 infection and negative test results on two nasopharyngeal and oropharyngeal swabs performed at least 24 h apart, testing of lower respiratory samples (bronchoalveolar lavage fluid obtained by bronchoscopy) was performed.

### Statistical analysis

We did not performed sample-size calculation. Patients were dichotomized into (1) patients with ischemic stroke and positive SARS-CoV-2 nucleic acid test (COVID-19), and (2) patients with ischemic stroke and negative test result (non-COVID-19). We then performed the following analyses:

*Exploratory analysis.* To characterize the study group continuous variables were reported as median (25th to 75th percentile) and categorical variables as number (%). For subgroup comparisons, we used the *χ*^2^ test or the Fisher exact test, and the Mann–Whitney *U* test, when appropriate.

#### Regression analysis

We performed a binary logistic regression analysis to identify pre-event variables associated to the status of COVID-19. The model included the following covariates: age, sex, traditional vascular risk factors, AF, malignancies, chronic kidney insufficiency, chronic obstructive pulmonary disease, and use of anti-platelets before stroke occurrence. Results are given as Odds Ratios (ORs) with 95% Confidence Intervals (CIs). Two-sided values of *p* < 0.05 were considered significant.

#### Survival analysis

Kaplan–Meier survival analysis was applied to estimate the incidence of in-hospital death by length of hospital stay of each participant, and multivariable Cox proportional hazards models to detect the parameters associated to in-hospital outcome. In both models time was expressed as length of hospital stay, from the index event until in-hospital death or hospital discharge. The following, prespecified potential predictors (variables with *p* < 0.05 in univariate comparison and potentially disease-modifying variables) were entered into the Cox models: COVID-19 status, pre-event mRS score, NIHSS score on admission, hypertension, smoking habit, atrial fibrillation, acute ischemic stroke treatment procedure, symptomatic intracranial hemorrhage, medical complications, recurrence of ischemic stroke during hospital stay, and sex and age which are important potential confounders. Results were expressed as Hazard Ratios (HRs) and 95% CIs. Two-sided values of *p* < 0.05 were considered significant.

Finally, we carried out Random Forests (RFs) classifier for the prediction of binary classification of in-hospital outcome (death vs survival) among patients with COVID-19 in the center where the patient was initially admitted. The RF is a classifier that includes a large number of decision tree classifiers. Each tree is trained with randomly selected with replacement (i.e., bootstrapped) learning samples and at each node a subset of features are randomly selected to generate the best split, and then the best classification is conducted based on a majority vote of the trees in the forest.

In addition, the bootstrap procedure naturally generates out-of-bag (OOB) samples. OOB subjects do not enter in the training process even if they belong to the initial training set. The entire process is called bagging. These OOB observations can be therefore used on-the-fly to estimate an OOB classification error that does not depend on either the training or the validation sets, giving an independent measure of classification reliability. Mean Decrease Accuracy (MDA), namely the importance of a feature in determining classification accuracy, and Mean Decrease Gini Impurity (MDG), namely the importance of a feature to discriminate between classes, were used for features ranking and plotting. In addition, multidimensional scaling (MDS) plot and the most representative tree plot were obtained for illustrative visualization of RF output [17].

#### Validation

To avoid classifier over-performance on a specific dataset, and consequent loss of classification generality and reproducibility, we performed a K-fold cross-validation analysis. At each iteration, K-1 partitions were merged into one and used for the learning process (training step), while the K-th left out partition (i.e., the validation set) was used to predict the outcome (i.e., death vs survival). Once every iteration cycle was completed, we moved to the next partitioning configuration, until every K-th partition has been used as validation set*.* We performed a multiple ten, five, and fourfold-cross validation analysis.

Performance of the classifier was computed considering by the followings indices [18]: (1) classification accuracy, i.e., the ratio of correctly predicted (positive or negative) observations to the total observations; (2) precision, i.e, the ratio of correctly predicted positive observations to the total predicted positive observations (precision estimates classifier’s ability to predict really positive observations when the test is positive); (3) recall, i.e., the ratio of correctly predicted positive observations to the total true positive observations (recall estimates the amount of true positive observations that were correctly classified as positive); and (4) F1-score, that is, the harmonic average of precision and recall.

The 2 × 2 frequency table (the so-called confusion matrix) was obtained at each iteration of the K-fold cross-validation, and the performance indices were computed both by averaging the indices of K 2 × 2 tables and using the overall 2 × 2 table over the K iterations. Descriptive and survival analyses were carried out using SPSS software (SPSS 21.0; Armonk, NY). Random forest classifier and evaluation of classification performance were carried out in R-4.0.02 [19] using *RandomForest* packagewith ntree = 1000 = number of trees to grow and mtry = sqrt(number of variables) randomly sampled as candidates at each split [20]; *reprtree* package, for selection of the most representative trees [21]; *CMA* package, for performance evaluation with K-fold cross-validation [22] and custom R functions for output visualization (MDS_plot, tree_plot).

## Results

A total of 1013 patients [median age, 76 years (25th to 75th) percentile, 18; males, 545 (53.8%)] qualified for the analysis. Of them, 853 (84.2%) had negative SARS-CoV-2 nucleic acid test and the remaining 160 (15.8%) had confirmed infection. The most common symptoms related to SARS-CoV-2 infection were fever (31.3%), cough (36.3%), dyspnoea (41.3%), and fatigue (25.6%). Stroke was the reason for hospital admission in all the patients. The laboratory diagnosis of infection was done a median of 2 days (25th to 75th percentile, 6.25) before stroke occurrence in 119 subjects based on typical symptoms (more frequently fever, cough, anorexia, and diarrhoea), while 41 were diagnosed at a later stage, during hospital stay [median, 2 days (25th to 75th percentile, 4)]. Fourteen patients of the latter group had positive test results after the first test on nasopharyngeal and oropharyngeal swab was negative, while seven had positive test result by the analysis of bronchoalveolar lavage fluid after two negative tests on nasopharyngeal and oropharyngeal swab. Since all patients in whom the diagnosis was made during hospital stay had had symptoms consistent with SARS-CoV-2 infection before admission, in addition to typical radiological signs in most cases, we assumed that all COVID-19 patients in the present series had their stroke after being infected.

### Patients with COVID-19 vs non-COVID-19 patients

Demographic and clinical characteristics of the study group, stratified by COVID-19 status, are summarized in Table 1, while standard diagnostic investigations are reported in Supplementary Table 1.

**Table 1:** Baseline demographic and stroke characteristics of the study group according to COVID-19 status

	COVID-19 (*n* = 160)	Non-COVID-19 (*n* = 853)	*p* value
Age, years	75.5 (66–83)	76 (65–82.75)	0.163
Sex, Male	94 (58.8)	451 (52.9)	0.172
Body Mass Index (kg/m^2^)	25.7 (23.6–27.8)	24.5 (22.6–27.5)	0.264
Hypertension	103 (64.4)	619 (72.6)	0.036
Diabetes	33 (20.6)	178 (20.9)	0.939
Hypercholesterolemia	47 (29.4)	293 (34.4)	0.218
Smoking habit			0.017
Never smoker	115 (75.7)	557 (67.8)	
Former smoker	24 (15.8)	117 (14.3)	
Current smoker	13 (8.6)	147 (17.9)	
Coronary heart disease	39 (24.4)	177 (20.8)	0.308
Atrial fibrillation	48 (30.0)	184 (21.6)	0.020
Personal history of ischemic stroke	17 (10.7)	124 (14.6)	0.197
Malignancies	18 (11.3)	77 (9.0)	0.376
Chronic kidney disease	9 (5.6)	43 (5.0)	0.699
Chronic obstructive lung disease	7 (4.4)	40 (4.7)	1.000
Pre stroke modified Rankin Scale (mRS)	1 (0–2)	0 (0–1)	0.001
Prior antiplatelets			0.026
1 antiplatelet	41 (25.9)	297 (34.8)	
2 antiplatelets	7 (4.4)	17 (2.0)	
Prior anticoagulants			0.201
Warfarin	17 (10.7)	60 (7.0)	0.091
DOACs	11 (6.9)	38 (4.5)	0.149
LMWH	1 (0.6)	5 (0.6)	0.896
Stroke severity, NIHSS score	9 (4–17)	6 (3–12)	≤ 0.001
Systolic blood pressure on admission, mm Hg	143 (130–160)	155 (140–170)	≤ 0.001
Dyastolic blood pressure on admission, mm Hg	80 (70–94)	80 (70–90)	0.418
Cause of stroke			0.001
Large-vessel disease	23 (14.4)	191 (22.4)	
Cardiac embolism	60 (37.5)	245 (28.7)	
Small-vessel disease	13 (8.1)	138 (16.2)	
Other determined etiology	7 (4.4)	52 (6.1)	
Undetermined etiology	57 (35.6)	227 (26.6)	
Acute stroke therapy			0.064
IV thrombolysis	17 (10.6)	108 (12.7)	
Endovascular thrombectomy	12 (7.5)	104 (12.2)	
IV thrombolysis and endovascular thrombectomy	8 (5.0)	73 (8.6)	
Standard medical treatment	123 (76.9)	568 (66.6)	

*DOACs* direct oral anticoagulants, *LMWH* low molecular weight heparin, *NIHSS* National Institute of Health Stroke Scale, *IV* intravenous

Patients with COVID-19 were less frequently smokers (current smokers, 8.6% vs 17.9%; OR 0.42; 95% CI 0.23–0.78) and hypertensive (64.4% vs 72.6%; OR 0.68; 95% CI 0.47–0.97). Although they were more often under treatment with anticoagulants before stroke occurrence (18.2% vs 12.1%; OR 1.62; 95% CI 1.03–2.55), cardioembolism was more frequently the presumed mechanism of brain ischemia (37.5% vs 28.7%, OR 1.48; 95% CI 1.04–2.11) and AF more frequently the underlying cause (30.0% vs 21.6%; OR 1.55; 95% CI 1.07–2.26) among patients with COVID-19 than among non-COVID-19 patients. At first clinical evaluation in the emergency room, patients with COVID-19 had higher NIHSS score [9 (25th to 75th percentile, 13) vs 6 (25th to 75th percentile, 9); OR 1.05; 95% CI 1.02–1.07] than non-infected stroke patients. On binary logistic regression analysis, male sex (OR 1.53; 95% CI 1.06–2.27) and AF (OR 1.60; 95% CI 1.05–2.43) were independently associated with COVID-19 status, whereas hypertension (OR 1.69; 95% CI 1.11–2.56) and active smoking (OR 2.56; 95% CI 1.33–5.00) were associated with non-COVID-19 status (Table 2).

**Table 2:** Binary logistic regression analysis showing the factors associated with COVID-19 in patients with acute ischemic stroke

	Adjusted OR (95% CI)	*p* value
Age	1.01 (0.99–1.03)	0.286
Sex, Male	1.53 (1.06–2.27)	0.024
Hypertension	0.59 (0.39–0.90)	0.014
Diabetes	1.09 (0.69–1.72)	0.725
Hypercholesterolemia	0.96 (0.63–1.47)	0.851
Smoking habit		
Never smoker	1	
Former smoker	0.94 (0.56–1.58)	0.817
Current smoker	0.39 (0.20–0.75)	0.004
Atrial fibrillation	1.60 (1.05–2.43)	0.027
Malignancies	1.20 (0.68–2.12)	0.536
Chronic kidney disease	1.09 (0.50–2.37)	0.829
Chronic obstructive lung disease	0.89 (0.37–2.12)	0.792
Prior antiplatelets	0.79 (0.52–1.21)	0.274

### In-hospital outcome

**Fig. 1:**
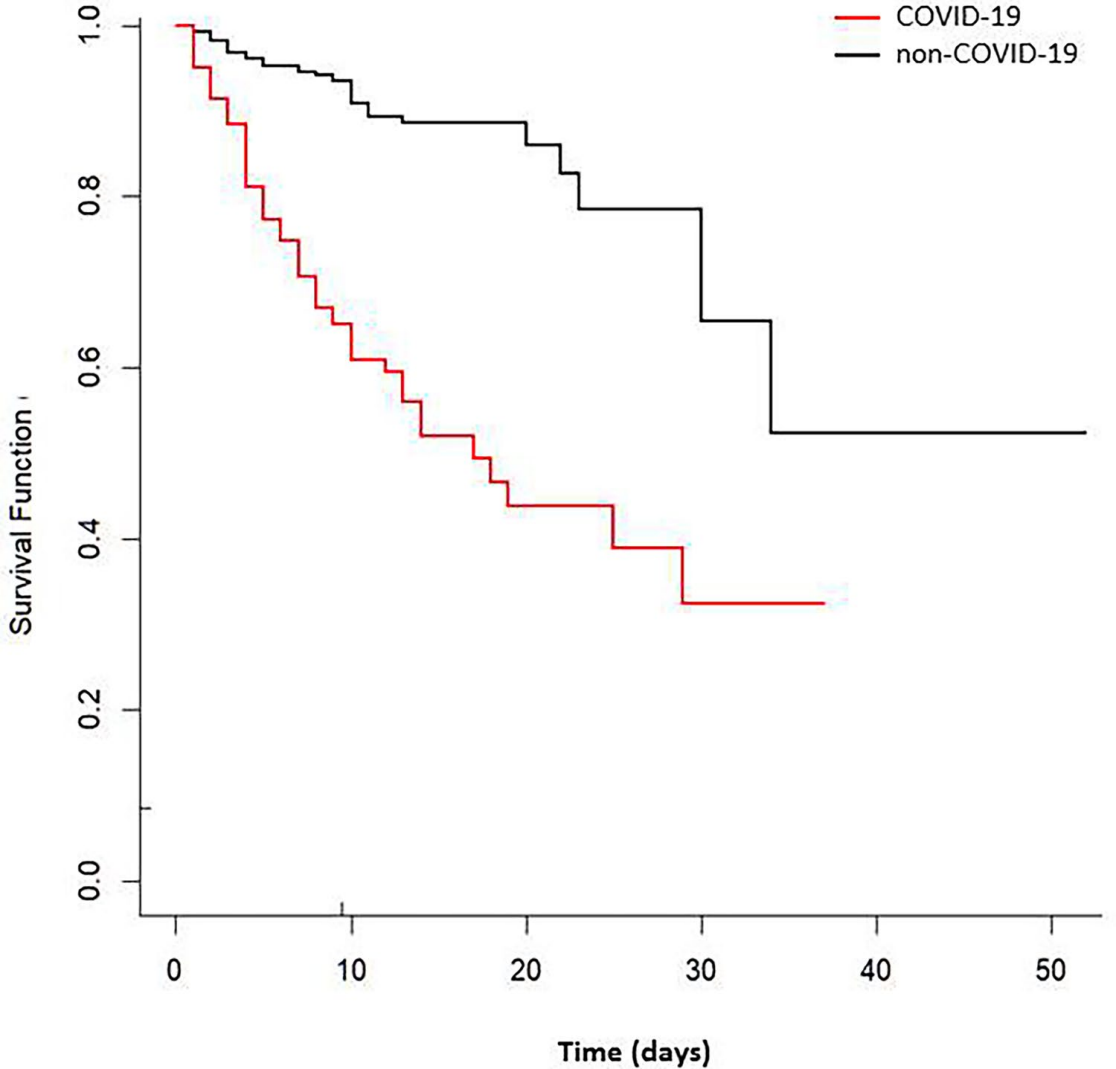
Kaplan–Meier estimates of in-hospital death by COVID-19 status in patients with acute ischemic stroke. Testing of significance is by the log-rank test

Ninety-four (58.8%) out of the 160 patients with confirmed infection received hydroxychloroquine, 97 (60.6%) antibiotics, 34 (21.3%) antivirals (lopinavir/ritonavir), and 23 (14.4%) corticosteroids. In 71 (44.3%) patients COVID-19 had a severe course. Ten (6.3%) patients with COVID-19 required invasive mechanical ventilation vs 17 (2.0%) non-COVID-19 patients (OR 3.27; 95% CI 1.47–7.29). Sixty-one (38.1%) subjects in the group of patients with COVID-19 died in hospital vs 61 (7.2%) in the group of non-COVID-19 patients (OR 8.00; 95% CI 5.30–12.07). The median interval between the index stroke and hospital discharge was 7 days (25th to 75th percentile, 5) vs 8 days (25^th^ to 75th percentile, 8) between the index stroke and in-hospital death (*p* = 0.026). In Kaplan–Meier analysis, SARS-CoV-2 infection was predictor of time until in-hospital death (*p* < 0.001, by the log-rank test; Fig. 1). The risk of death at 10 days after stroke onset was approximately 40% for patients with confirmed SARS-CoV-2 infection versus 5% for patients without infection.

Prespecified multivariable Cox regression models demonstrated that SARS-CoV-2 infection was independently associated with increased risk of in-hospital death, and this effect remained consistent in multivariable models controlling for different sets of variables (Table 3).

**Table 3:** Multivariable analysis of predictors of in-hospital death in patients with acute ischemic stroke

Variables	HR (95% CIs)	*p* value
SARS-CoV-2 infection	3.30 (2.17–5.02)	≤ 0.001
Age	1.03 (1.00–1.05)	0.017
Sex, female	0.62 (0.39–1.00)	0.049
Pre stroke modified Rankin Scale (mRS)	0.95 (0.80–1.13)	0.567
Hypertension	0.87 (0.56–1.37)	0.554
Atrial fibrillation	1.14 (0.70–1.86)	0.591
Smoking habit		
Never smoker	1	
Former smoker	1.00 (0.56–1.78)	0.994
Current smoker	0.94 (0.45–1.93)	0.857
NIHSS score	1.10 (1.06–1.14)	≤ 0.001
Recanalizing therapy	0.76 (0.47–1.17)	0.211
Symptomatic cerebral hemorrhage	6.90 (2.86–16.61)	≤ 0.001
Medical complications	3.99 (2.48–6.41)	≤ 0.001
Ischemic stroke recurrence	0.56 (0.16–1.99)	0.371

*HR* hazard ratio, *CI* confidence interval, *NIHSS* National Institute of Health Stroke Scale

### RF models, and classification performances

**Fig. 2:**
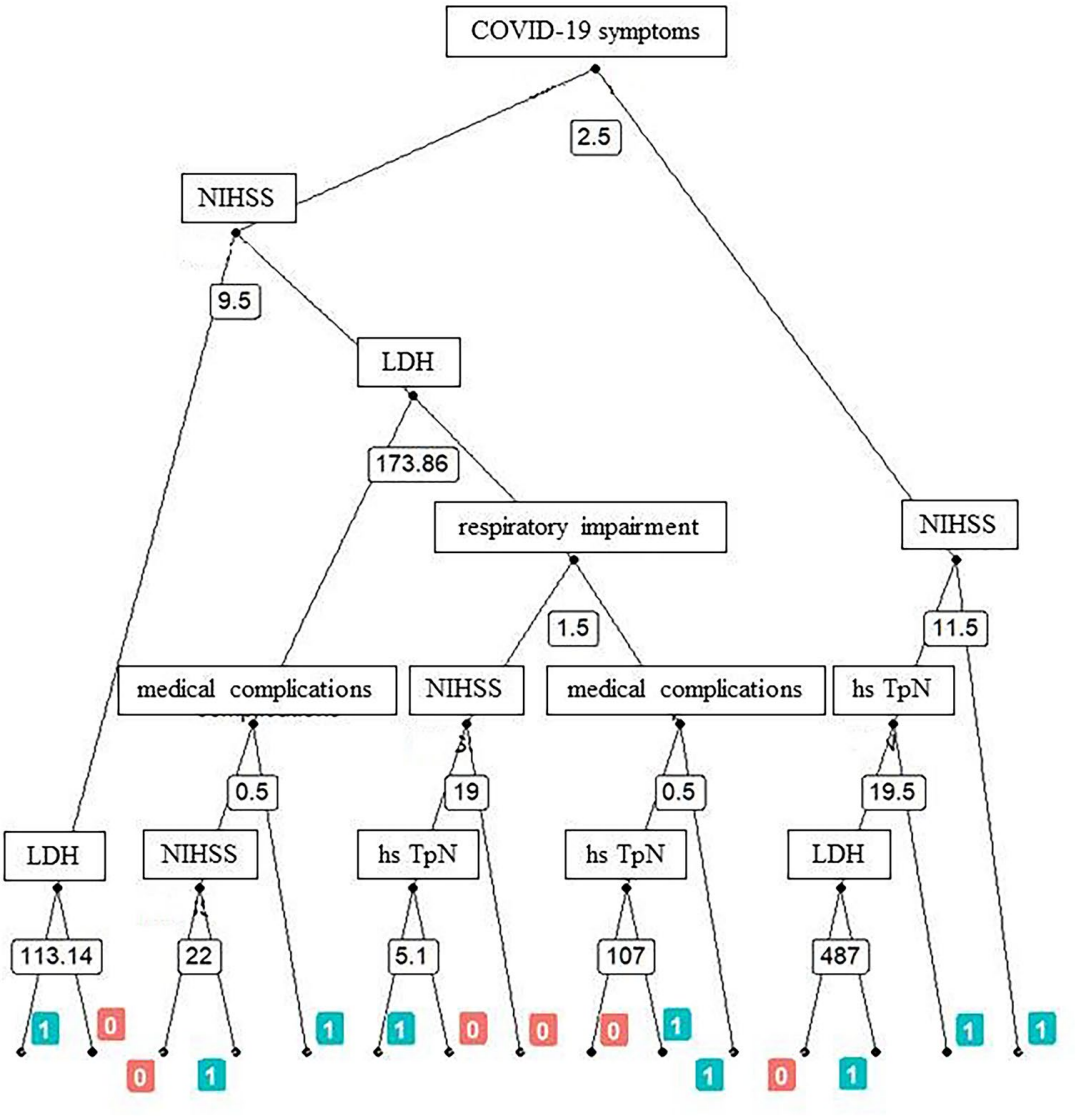
The most representative tree plot of the 1000 trees used in the RF model. The tree visualizes a clear separation between ischemic stroke patients with COVID-19 who died in hospital and ischemic stroke patients with COVID-19 who survived, with the root node defined by the presence of COVID-19 symptoms at hospital admission. *NIHSS* National Institute of Health Stroke Scale, *LDH* lactate dehydrogenase, *hs* *TpN* high sensitivity cardiac troponin. COVID-19 symptoms and medical complications were binary variables

Clinical characteristics and laboratory findings of patients with COVID-19, stratified by in-hospital outcome, are presented in Supplementary Tables 2 and 3. For each of the 160 patients a set of 30 clinical and laboratory parameters was retained, and a first RF model was performed. The six top-ranking parameters providing the best classification accuracy and discrimination of in-hospital outcome (death vs survival) were the following: (1) COVID-19 symptoms, (2) severity of respiratory impairment, (3) plasma levels of high-sensitivity cardiac troponin at admission, (4) NIHSS score at admission, (5) occurrence of medical complications, and (6) serum levels of lactate dehydrogenase (LDH) at admission (Supplementary Fig. 2). These parameters were used in the subsequent 2-group RF classifier. Multidimensional scaling (MDS) plots for this classifier are shown in Supplementary Fig. 3, while the most representative tree plot of the 1,000 trees used in the RF model is shown in Fig. 2. The tree clearly shows how the selected parameters and their values can predict in-hospital outcome at individual level. In the 2-group classifier, classification indices with fivefold cross-validation (ten- and fourfold cross-validation produced similar results) and outliers deletion (with Brier score > 1) on 136 patients with COVID-19 indicated that the prediction model had overall high accuracy (0.86) and precision (0.87), as well as good recall (0.72) and F1-score (0.79).

## Discussion

In this large collaborative study of patients with acute ischemic stroke conducted in Lombardy, a region hard-hit by SARS-CoV-2 outbreak, infection was a frequent finding, affecting approximately 16% of patients, with obvious consequences in terms of in-hospital survival and disability. Furthermore, the present report provides detailed information on the specific characteristics of acute ischemic stroke patients with confirmed infection, which might have clinical implications. As reported in previous studies involving patients with acute ischemic stroke, male sex was associated with infection [8]. Furthermore, compared to SARS-CoV-2 negative patients, patients with COVID-19 more often had cardioembolism, especially related to AF, as likely cause of brain ischemia and a lower burden of conventional cardiovascular risk factors, in particular hypertension [8] and smoking.

Although the elucidation of the exact underlying process falls beyond the scope of our study, there is a clear biological plausibility for these findings. Male sex has been repeatedly associated to worse outcomes in patients with COVID-19 compared to female sex, and a number of mechanisms have been proposed to explain such a sex-differential risk. These include differences in immunological response related to sex chromosomes, higher levels of antibodies in women relative to men [23, 24], and hormonal differences in susceptibility to coronaviruses infection [25]. It is likely that the processes involved in sex-related COVID-19 severity may also favor the occurrence of extrapulmonary complications, including ischemic stroke, in men more than in women.

Acute infections, more often pneumonia or bronchitis, are well-known and frequent precipitants of incident AF, being responsible for approximately one fifth of secondary cases [26], though there is still uncertainty, when it comes to thromboembolic risk, as to whether AF due to reversible precipitants differs from non-secondary cases. Growing data have proposed inflammation as the most likely mechanism linking infection to induction and maintenance of AF [27, 28]. Cytokine activation can trigger atrial arrhythmias in patients who develop acute myocarditis or inflammatory response and in those with underlying coronary or other structural heart diseases. Early reports from China suggested an overall cardiac arrhythmia incidence of ~ 17% in hospitalized COVID-19 patients, and a higher arrhythmia rate (~ 44%) in COVID-19 patients admitted to the intensive care unit. However, no definition as to what constituted an arrhythmia was provided [29]. Similarly, incident AF was detected in ~ 3.6% of patients with COVID-19 and no previous history of cardiac arrhythmias [30]. The common occurrence of cardiac arrhythmias in patients with COVID-19, as opposed to what reported for coronavirus infections due to SARS and Middle East respiratory syndrome (MERS) [31, 32], raises a question about SARS-CoV-2-specific mechanisms in the pathogenesis of AF. Regardless of the mechanisms linking SARS-CoV-2 infection to AF, it is clear that inflammation is intimately linked to a prothrombotic state and, hence, to thrombogenesis in patients with AF [27]. Additionally, hypoxia and its downstream signalling, common complications of COVID-19, also promote thrombus formation and propagation [33]. The mechanisms underlying thrombogenesis in AF are complex and only partly understood, however, it appears likely that SARS-CoV-2 infection might trigger AF in susceptible individuals, promote thrombogenesis in these subjects and in those with pre-existing AF, and increase the propensity to thromboembolic events [34], especially ischemic stroke. Besides their biological implications, these consistent findings provide further arguments in favor of therapeutic anticoagulation to prevent thromboembolic complications, including ischemic stroke, in selected patients with COVID-19.

As to the lower burden of conventional cardiovascular risk factors in patients with SARS-CoV-2 infection, it is plausible that the underlying inflammatory-prothrombotic state observed in these patients increases individual sensitivity to ischemia and predisposes to stroke occurrence even in the absence of further susceptibility conditions.

Another, not unexpected [4, 7, 8], finding of the present study was that patients with acute ischemic stroke who were COVID-19 positive had increased stroke severity at admission and much higher in-hospital mortality rate than non-infected patients. The reason why COVID-19-related brain infarctions are characterized by increased stroke severity remains largely speculative. Theoretically, infection-induced systemic inflammatory response might play a role, as it causes accumulation of inflammatory cells in the vascular wall, increased blood–brain barrier permeability, endothelial dysfunction and, as an end result, impaired automatic regulation of cerebral circulation [3]. This might promote rapid infarct expansion into tissue with milder perfusion deficits and diminish salvageable tissue at risk (i.e., ischemic penumbra). AF itself is associated with increased stroke severity [35]. The higher rate of large-artery occlusion which has been observed in the group SARS-CoV-2 positive patients with acute ischemic stroke [36], though presently unconfirmed, might also contribute. Similarly, whether the underlying prothrombotic state of COVID-19 patients is also partly responsible is an attractive but untestable hypothesis in the setting of the present study. Whatever the reason, the increased stroke severity at admission in patients with COVID-19 is a major contributor to the higher in-hospital mortality rate observed in these patients, as confirmed by the results of the machine-learning-based computer-aided analysis of data within the group of patients with confirmed SARS-CoV-2 infection. Advanced machine learning, a subfield of artificial intelligence, is increasingly applied to biomedical and health-care data for the development of early diagnostic tools. The clinical implications of our findings is that a simple model based on clinical and routine laboratory parameters is possibly able to selectively identify patients with acute ischemic stroke and confirmed SARS-CoV-2 infection who are unlikely to survive the acute phase and distinguish them from those who will.

### Strengths and limitations

The strength of the present work relies on the large sample size, with a multicenter, consecutive enrollment, and the machine-learning computer-aided approach to data analysis to obtain the highest possible values of accuracy in predicting in-hospital outcome. In this regard, the sample size allowed us to apply a fivefold cross-validation, which prevents results from overfitting and guarantees replicability to other datasets. Moreover, the RF classifier is superior to most available learning algorithms, because it is easy to parameterize, robust against overfitting, not sensitive to noise in the dataset (i.e., good at dealing with outliers in training data), and able to avoid biases due to unrelated centers [37]. A potential limitation of this approach is that findings should be replicated in an independent set of subjects with comparable demographic, clinical and laboratory characteristics, to assess their generalizability. Because of the specific setting of the STROKOVID project, however, such an independent set is obviously difficult to get.

Another methodological advantage of our study is the inclusion of contemporaneous non-COVID-19 stroke controls, rather than historical stroke controls. Historical controls cannot be relied on to determine how COVID-19 might affect the phenotype of stroke since the pattern of stroke overall, regardless of COVID-19, is greatly influenced by the pandemic (due to changes in stroke pathways, hospital admission strategies and patient behavior). Our approach allows, therefore, to minimize potential selection bias when comparing COVID-19 patients with non-COVID-19 patients.

Notwithstanding, the results of our study should be interpreted in the context of its observational, retrospective design. The following limitations should be considered. First, it may be that not all cases of acute ischemic stroke in the catchment area were included in our cohort, because this is a hospital-based study, rather than a community-based study. Those patients who sustained a fatal stroke, had minor stroke symptoms, did not attend emergency departments due to fear of acquiring the infection or simply to avoid adding to an overburdened healthcare system, were not admitted and, thus, would not have been included in the present analysis. However, this is an unavoidable feature of all hospital-based studies. Furthermore, since the STROKOVID network, unlike other studies [4, 8], includes all centers designated as hubs for the management of acute stroke in a given geographical area, we can assume that all acute strokes that occurred in Lombardy during the study period were included in the present analysis, which makes any further selection bias very unlikely. In addition, as the general characteristics of our cohort of non-infected patients reflect those of other hospital series of ischemic stroke patients [38, 39], we presume that our study population is a representative sample of Italian patients with acute ischemic stroke. Second, it is possible that the non-systematic ascertainment of AF in our patients may have resulted in misclassification, especially for asymptomatic paroxysms. However, though we do not dispute that a systematic cardiac monitoring would have allowed us to diagnose more AFs, it is unlikely that this have influenced our results. Actually, if there were any effect, this would have affected the group of COVID-19 patients, who were less frequently monitored, more than the group of non-COVID-19 patients, thus providing further strength to our findings. Third, investigators were, unavoidably, not blinded to the COVID-19 status of the patients. This could theoretically implicate biases in the analysis, such as, for example, misclassification of etiological categories of ischemic strokes. However, all the variables we used in the analysis, including TOAST classification, were defined according to validated criteria, which makes us confident of the accuracy of our data collection and any potential shortcomings resulting from the non-blinded status of the raters very unlikely. Forth, some of the diagnoses in the local registries may have different accuracy during a public health crisis, which should be kept in mind when evaluating the results of a multicenter study. Finally, although we were able to adjust for multiple confounding factors, we cannot rule out unmeasured or residual confounding, given the observational nature of our study. This is the case, for example, of the performances of stroke team members in complying with personal protection equipment (PPE, masks, eye protection, gowns, and gloves) and other infection control recommendations, especially under extreme situations, such as those observed in Lombardy in the early stages of the epidemic, which might have had an impact on patients outcome, or of the redeployment of stroke neurologists to other settings to respond to the increasing demands of COVID-19 in some centres, which might have created a significant gap in care.

## Conclusions

Our study showed that acute ischemic stroke is a frequent complication of SARS-CoV-2 infection and it is more often the consequence of cardiac sources of embolism, especially AF. Patients with confirmed infection have increase stroke severity and the rate of in-hospital death far exceeds that among non-COVID-19 patients. A simple model based on clinical and routine laboratory parameters may be an additional screening tool to be used in clinical practice to predict in-hospital outcome of these patients.

## Supplementary Information

Below is the link to the electronic supplementary material.

Supplementary file1 (DOC 444 KB)

Supplementary file2 (DOC 100 KB)

Supplementary file3 (DOCX 96 KB)

## Data Availability

The data that support the findings of this study are available from the corresponding author on reasonable request.
