# Supplementary material for: SARS-CoV-2 infection and acute ischemic stroke in Lombardy, Italy
Source: J Neurol. 2021 May 24;269(1):1–11. doi: 10.1007/s00415-021-10620-8 (PMC8142879; doi:10.1007/s00415-021-10620-8)
Supplement: Supplementary file 1 — Supplementary file1 (DOC 444 KB) [file 415_2021_10620_MOESM1_ESM.doc]

**Supplementary Material**

**SARS-CoV-2 infection and acute ischemic stroke in Lombardy, Italy**

Alessandro Pezzini et al.

**Supplementary materials & methods**

**Table 1**

**Standard diagnostic work-up according to COVID-19 status**

**Table 2**

**Clinical characteristics of COVID-19 patients stratified by in-hospital outcome**

IQR, interquartile range; NIHSS, National Institute of Health Stroke Scale; IV, intravenous; ARDS, acute respiratory distress syndrome. *Fever was defined as axillary temperature of at least 37.3°C.

**Table 3**

**Laboratory findings in COVID-19 patients stratified by in-hospital outcome**

ALT, alanine aminotransferase

**Figure 1**

**Hub centers participating to the STROKOVID collaborative research project**.

Carlo Poma Hospital (Mantova, Italy), Spedali Civili, University Hospital (Brescia, Italy), San Gerardo, University Hospital (Monza, Italy), Ospedale Nuovo (Legnano, Italy), Niguarda (Milano, Italy), di Circolo e Fondazione Macchi Hospital (Varese, Italy), “A. Manzoni” Hospital (Lecco, Italy), IRCCS Humanitas Clinical and Research Hospital (Rozzano-Milano, Italy), Sant’Anna Hospital (Como, Italy), IRCCS Fondazione “C. Mondino” Hospital (Pavia, Italy).

**Figure 2**

**Mean Decrease Accuracy (MDA) and Mean Decrease Gini (MDG) Impurity of the first run of the RF model including 30 predictive variables.**

TpN, high sensitivity cardiac troponin; NIHSS, National Institute of Health Stroke Scale; LDH, lactate dehydrogenase; INR, International Normalized Ratio; CRP, C reactive protein; DOACs, direct oral anticoagulants; CPK, creatine phosphokinase; ICH, intracerebral haemorrhage; aPTT, activated partial thromboplastin time; ALT, alanine aminotransferase.

**Figure 3**

**Multidimentional scaling (MDS) plot according to the two-group RF classifier (Y = 1, ischemic stroke patients with COVID-19 who died in hospital; Y = 0, ischemic stroke patients with COVID-19 who survived) using the selected 6 top-predictive variables.**

MDS plots visualize the proximity matrix accumulated for the training data by out-of-bag observations, that is, predicted values for each observation in the training dataset that are not included in the bootstrap samples. MDS plots tend to form a separate shape: the most pronounced the separation, the better the classification performance that is obtained.

**List of collaborators**

**Supplementary materials & methods**

*Risk factor definition*

Hypertension was defined as systolic blood pressure 140 mm Hg or higher and diastolic pressure 90 mm Hg or higher in 2 separate measurements after the acute phase or use of antihypertensive drugs before recruitment; diabetes, with a history of diabetes, use of a hypoglycemic agent or insulin, or fasting glucose level 126 mg/dL or higher; current smoking, including former smokers who had quit smoking for 2 years before the index event; hypercholesterolemia, with cholesterol serum levels 220 mg/dL or higher or use of cholesterol lowering drugs. Body mass index (BMI) was calculated as weight in kilograms divided by the square of the height in meters (kg/m^2^). All patients underwent continuous cardiac monitoring using standard bedside monitors immediately after SU admission. In those patients who had not received a diagnosis before the index stroke, atrial fibrillation (AF) was eventually diagnosed by a cardiologist based on the ECGs performed in the emergency room with patients in a supine position using standard, 10-second, 12-lead ECG devices, as well as on the ECG recordings performed during hospital stay. We also collected information on history of coronary ischemic heart disease (myocardial infarction, history of angina, or existence of multiple lesions on thallium heart isotope scan or evidence of coronary disease on coronary angiography), previous ischemic stroke (based on clinical history or medical records), and pre-stroke medications (in particular, warfarin, aspirin or other antiplatelet agents, antihypertensive agents, oral hypoglycemic agents or insulin, and statins).

Chronic obstructive pulmonary disease (COPD) was diagnosed based on the Global Initiative for Chronic Obstructive Lung Disease (GOLD) criteria [1]. Chronic kidney disease (CKD) was defined according to the 2012 KDIGO clinical practice guidelines [2]. Functional status before stroke occurrence was measured by the modified Rankin scale (mRS) [3].

*Clinical Assessment*

The 4 groups categorized based on the severity of respiratory impairment were defined as follows: 1) mild type: mild clinical symptoms without pneumonia in imaging; 2) common type: fever, respiratory tract and other symptoms with pneumonia in imaging; 3) severe type: respiratory distress, respiratory rate ≥30 times/min; in resting state, oxygen saturation ≤93%; PaO2/FiO2 ≤300 mm Hg; 4) critical type: respiratory failure requiring mechanical ventilation, shock and other organ failure requiring ICU monitoring and treatment.

*Medical complications*

Sepsis and septic shock were defined according to the 2016 Third International Consensus Definition for Sepsis and Septic Shock [4,5]; 2) secondary infection was diagnosed when patients show clinical symptoms or signs of pneumonia or bacteraemia and a positive culture of a new pathogen was obtained from lower respiratory tract specimens (qualified sputum, endotracheal aspirate, or bronchoalveolar lavage fluid) or blood samples after admission [6]; 3) acute respiratory distress syndrome (ARDS), was diagnosed according to the Berlin Definition [7]; 4) acute kidney injury was diagnosed according to the KDIGO clinical practice guidelines [8]; 5) acute cardiac injury was diagnosed if serum levels of cardiac biomarkers (eg, high sensitivity cardiac troponin I) were above the 99^th^ percentile upper reference limit, or if new abnormalities were shown in electrocardiography and echocardiography; 6) coagulopathy was defined as a 3-second extension of prothrombin time (PT) or a 5-second extension of activated partial thromboplastin time (aPTT); 7) hypoproteinaemia was defined as blood albumin of less than 25 g/L; and symptomatic intracranial hemorrhage was defined according to the Second European-Australasian Acute Stroke Study (ECASS-II) criteria, as blood at any site in the brain on the CT scan, documentation by the investigator of clinical deterioration, or adverse events indicating clinical worsening (eg, drowsiness, increase of hemiparesis) or causing an increase in the NIHSS score of 4 or more points [9].

**Table 1**

**Table 2**

**Table 3**


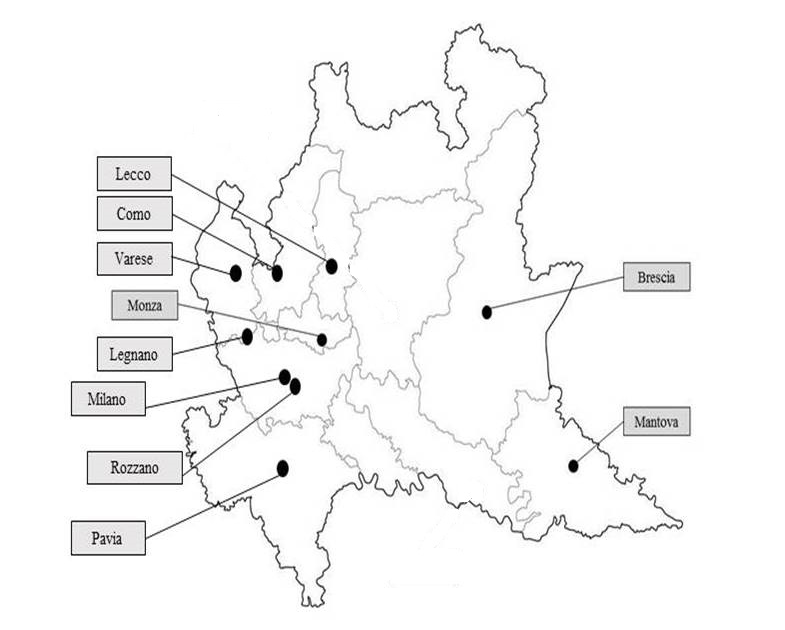


**Figure 1**


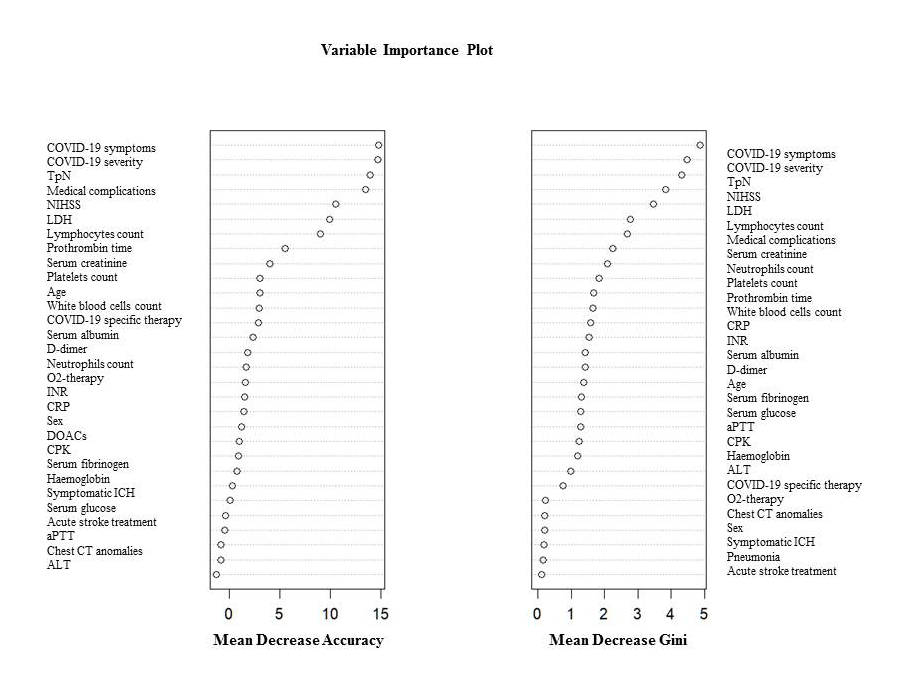


**Figure 2**


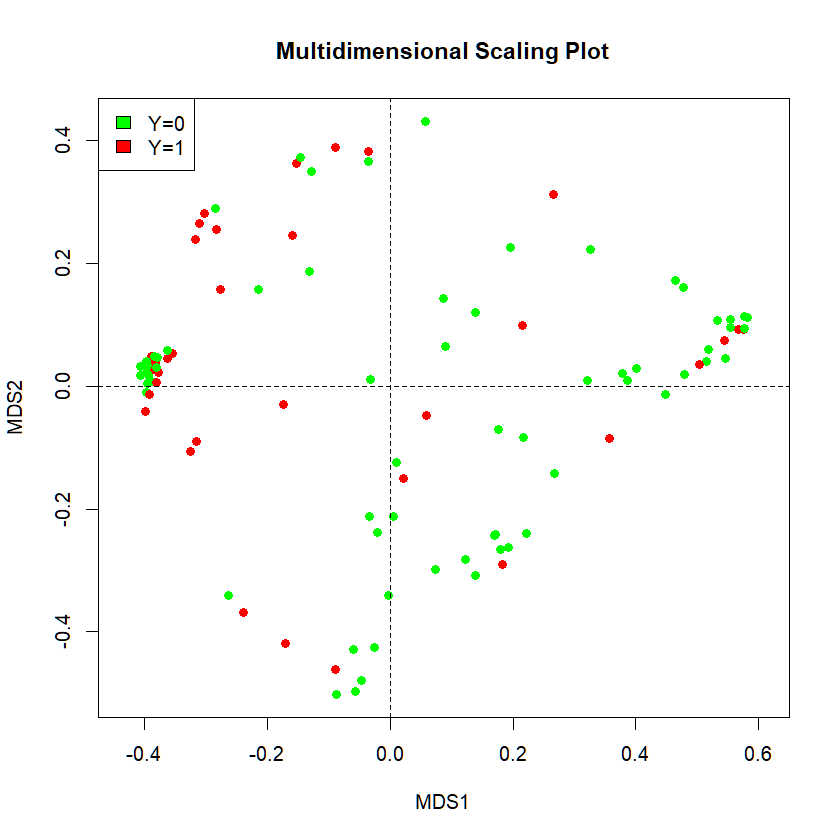


**Figure 3**

**List of collaborators (by participating centre):**

*Bergamo*

Department of Neurology, Papa Giovanni XXIII Hospital: Dario Alimonti, Emanuela Agazzi, Giorgia Camera, Virginio Bonito, Camillo Foresti, Barbara Frigeni, Maria Grimoldi,

Stefano Quadri, Riccardo Riva, Elisabetta Venturelli, Tania Parziguian, Marcella Vedovello,

Marta Zaffira Conti, Manlio Sgarzi.

Department of Neuroradiology, Papa Giovanni XXIII Hospital: Gabriele Gallizioli

*Brescia*

Department of Clinical and Experimental Sciences, Neurology Clinic, University of Brescia:

Chiara Agosti, Antonella Alberici, Enrico Baldelli, Sonia Bonacina, Barbara Borroni,

Salvatore Caratozzolo, Stefano Cotti Piccinelli, Elisabetta Cottini, Viviana Cristillo,

Stefano Gipponi, Marcello Giunta, Alberto Imarisio, Paolo Liberini, Ilenia Libri, Debora Pezzini, Loris Poli, Renata Rao, Barbara Risi, Luca Rozzini, Andrea Scalvini, Francesca Schiano di Cola, Irene Volonghi, Nicola Zoppi.

Vascular Neurology – Stroke Unit, Spedali Civili Hospital, ASST Spedali Civili: Angelo Costa, Ilenia Delrio, Nicola Gilberti, Raffaella Spezi, Veronica Vergani

Neurophysiology Unit, ASST Spedali Civili: Stefano Gazzina, Ugo Leggio.

Department of Neuroimmunology and Neuromuscular Diseases, Foundation IRCCS Neurological Institute Carlo Besta, Milan: Laura Brambilla

Neurology Unit, School of Medicine, University of Bologna, Bologna: Matteo Benini

Neuroradiology Unit, University of Brescia: Roberto Gasparotti, Dikran Mardighian,

Michele Frigerio.

*Cremona*

Neurology Unit, Istituti Ospitalieri: Francesca Caprioli, Monica Monchini, Chiara Campana,

Chiara Costanzi, Matilde Milani, Francesca Tamburelli, Gloria Maccabelli.

Neuroradiology Unit, Istituti Ospitalieri: Michele Besana, Claudia Ambrosi.

*Mantova*

Department of Neurology and Stroke Unit, Carlo Poma Hospital: Mirko Avesani, Luca Bertolani, Maria Angela Bonometti, Francesca Girolami, Marco Marchini, Saverio Silipo,

Carlo Maria Stucchi, Monica Tottola, Carmela Zuco.

Department of Neurology and Stroke Unit, Carlo Poma Hospital: Enrico Piovan, Carlo Sozzi, Mirko Trentadue.

*Monza*

Department of Neurology, Ospedale San Gerardo, ASST Monza, Department of Medicine and Surgery and Milan Center for Neuroscience, University of Milano-Bicocca: Martina Viganò, Fiammetta Pirro, Claudia Cutellè, Benedetta Storti, Claudia Balducci, Francesco Pasini,

Danilo Antonio Montisano, Maria Luisa Piatti.

*Lecco*

Neurology Unit, Presidio A. Manzoni: Chiara Scaccabarozzi, Angelo Aliprandi,

Gisella Costantino.

Emergency Department, Presidio A. Manzoni: Luciano D'Angelo, Domenico Colombo

Neuroradiology Unit, Presidio A. Manzoni: Alessandro Lunghi, Marco Giuseppe Filizzolo.

*Pavia*

Stroke Unit, IRCCS Fondazione Mondino, Pavia: Alessandra Persico, Federico Mazzacane,

Piera Tosi, Serena Magno, Mariagiovanna Cuzzoni, William Boadu, Gianpaolo Toscano,

Maria Federica Denaro.

Neuroradiology Unit, IRCCS Policlinico San Matteo Pavia: Elvis Lafe, Alessandro Sgreggia, Giuseppina Sanfilippo.

**Supplemental references**

1. [Vogelmeier](https://pubmed.ncbi.nlm.nih.gov/?sort=date&term=Vogelmeier+CF&cauthor_id=28128970)CF, [Criner](https://pubmed.ncbi.nlm.nih.gov/?sort=date&term=Criner+GJ&cauthor_id=28128970) GJ, [Martinez](https://pubmed.ncbi.nlm.nih.gov/?sort=date&term=Martinez+FJ&cauthor_id=28128970) FJ, [Anzueto](https://pubmed.ncbi.nlm.nih.gov/?sort=date&term=Anzueto+A&cauthor_id=28128970)A, [Barnes](https://pubmed.ncbi.nlm.nih.gov/?sort=date&term=Barnes+PJ&cauthor_id=28128970) PJ, [Bourbeau](https://pubmed.ncbi.nlm.nih.gov/?sort=date&term=Bourbeau+J&cauthor_id=28128970) J, Celli BR, Chen R, Decramer M, Fabbri LM,  et al. Global strategy for the diagnosis, management, and prevention of chronic obstructive lung disease 2017 Report. GOLD Executive Summary. Am J Respir Crit Care Med. 2017;195:557-582.
2. Kidney Disease: Improving Global Outcomes (KDIGO) CKD Work Group. KDIGO 2012 Clinical practice guideline for the evaluation and management of chronic kidney disease. Kidney Int Suppl. 2013;3:1-150.
3. van Swieten JC, Koudstaal PJ, Visser MC, Schouten HJ, van Gijn J. Interobserver agreement for the assessment of handicap in stroke patients. Stroke. 1988;19:604-607.
4. Seymour CW, Liu VX, Iwashyna TJ, Brunkhorst FM, Rea TD, Scherag A, Rubenfeld G, Kahn JM, Shankar-Hari M, Singer M, et al. [Assessment of clinical criteria for sepsis: for the Third International Consensus Definitions for Sepsis and Septic Shock (Sepsis-3).](https://www-ncbi-nlm-nih-gov.proxy.unibs.it/pubmed/26903335) JAMA. 2016;315:762-74.
5. [Shankar-Hari M,](https://jamanetwork-com.proxy.unibs.it/searchresults?author=Manu+Shankar-Hari&q=Manu+Shankar-Hari)[Phillips GS,](https://jamanetwork-com.proxy.unibs.it/searchresults?author=Gary+S.+Phillips&q=Gary+S.+Phillips)[Levy ML,](https://jamanetwork-com.proxy.unibs.it/searchresults?author=Mitchell+L.+Levy&q=Mitchell+L.+Levy) [Seymour](https://pubmed.ncbi.nlm.nih.gov/?sort=date&term=Seymour+CW&cauthor_id=26903336)  CW,  [Liu](https://pubmed.ncbi.nlm.nih.gov/?sort=date&term=Liu+VX&cauthor_id=26903336) VX, [Deutschman](https://pubmed.ncbi.nlm.nih.gov/?sort=date&term=Deutschman+CS&cauthor_id=26903336) CS,  [Angus](https://pubmed.ncbi.nlm.nih.gov/?sort=date&term=Angus+DC&cauthor_id=26903336) DC, [Rubenfeld](https://pubmed.ncbi.nlm.nih.gov/?sort=date&term=Rubenfeld+GD&cauthor_id=26903336) GD, [Singer](https://pubmed.ncbi.nlm.nih.gov/?sort=date&term=Singer+M&cauthor_id=26903336) M. Developing a New Definition and Assessing New Clinical Criteria for Septic Shock For the Third International Consensus Definitions for Sepsis and Septic Shock (Sepsis-3) JAMA. 2016;315:775-87.
6. Huang C, Wang Y, Li X, , [Ren](https://pubmed.ncbi.nlm.nih.gov/?sort=date&term=Ren+L&cauthor_id=31986264) L, [Zhao](https://pubmed.ncbi.nlm.nih.gov/?sort=date&term=Zhao+J&cauthor_id=31986264) J, [Hu](https://pubmed.ncbi.nlm.nih.gov/?sort=date&term=Hu+Y&cauthor_id=31986264) Y, [Zhang](https://pubmed.ncbi.nlm.nih.gov/?sort=date&term=Zhang+L&cauthor_id=31986264) L, [Fan](https://pubmed.ncbi.nlm.nih.gov/?sort=date&term=Fan+G&cauthor_id=31986264) G, [Xu](https://pubmed.ncbi.nlm.nih.gov/?sort=date&term=Xu+J&cauthor_id=31986264) J, [Gu](https://pubmed.ncbi.nlm.nih.gov/?sort=date&term=Gu+X&cauthor_id=31986264) x, et al. Clinical features of patients infected with 2019 novel coronavirus in Wuhan, China. Lancet 2020;395:497–506.
7. The ARDS Definition Task Force*Acute Respiratory Distress Syndrome. The Berlin Definition. JAMA. 2012;307(23):2526-2533
8. Khwaja A. KDIGO clinical practice guidelines for acute kidney injury. Nephron Clin Pract 2012;120:c179–84
9. Hacke W, Kaste M, Fieschi C, von Kummer R, Davalos A, Meier D, Larrue V, Bluhmki E, Davis S, Donnan G,  et al. Randomised double-blind placebo-controlled trial of thrombolytic therapy with intravenous alteplase in acute ischaemic stroke (ECASS II). Second European-Australasian Acute Stroke Study Investigators. Lancet 1998;352:1245-1251
